# Thermo-Mechanical Simulation of Hybrid Welding of DP/AISI 316 and TWIP/AISI 316 Dissimilar Weld

**DOI:** 10.3390/ma13092088

**Published:** 2020-05-01

**Authors:** Patrizia Perulli, Michele Dassisti, Giuseppe Casalino

**Affiliations:** Department of Mechanics Management Mathematics—DMMM, Polithecnic of Bari, via Orabona 4, 70125 Bari, Italy; patrizia.perulli@poliba.it (P.P.); michele.dassisti@poliba.it (M.D.)

**Keywords:** dissimilar metals, laser welding, numerical simulation

## Abstract

In this paper, hybrid laser-MAG (metal active gas) welding of twinning-induced plasticity (TWIP) and dual-phase (DP) steels with austenitic stainless steel (AISI316) was simulated by means of the finite element method. A thermo-mechanical model, which uses a 3D heat sources, was developed using the software Simufact Welding. The calculated dimensions, shape and distortion of the weld were compared with the experimental results, thence the model was validated. The metallurgical transformations for the DP steel were evaluated using the continuous cooling transformation (CCT) diagram and the calculated cooling rate. The numerical model predicted accurately the shape of the molten pool, the thermal cycles as well as the geometrical distortion of the butt weld. Therefore, the numerical model showed a good reliability and its potential for further development.

## 1. Introduction

Nowadays, the increased interest of manufacturing industry in welding is due to its great economical as well as technological advantages such as higher operating efficiency and reduction in fabrication cost. Hybrid welding shows a great future compared to stand-alone laser welding and arc welding techniques. Laser welding coupled with arc welding offers several advantages like increasing depth of weld, wider gap tolerance, higher welding speeds, narrow heat-affected zone (HAZ) and reduced welding defects [1,2]. There are many applications that have proved hybrid laser-arc welding to be a suitable technology to weld metals of different compositions and properties [3].

The combination of different materials is often required to provide high-performing products [4]. Among steels, high manganese twinning-induced plasticity (TWIP) steels and dual phase (DP) can be joined to austenitic stainless steels and they produce high strength, although high corrosion and oxidation resistance welds [5,6,7,8,9,10]. TWIP steel with austenitic microstructures at room temperature show high strength and ductility due to the formation of twins during deformation. DP steel, with a ferritic and martensitic microstructure, shows high tensile strength (from 500 to 1200 MPa) and formability. The formation of martensite microstructure increases the tensile strength [3]. Dissimilar joints must have adequate tensile strength and ductility, which requires avoiding the formation of unsuitable microstructures and second phases in the fusion and HAZ [11]. Through finite element analysis, it is possible to develop time-saving numerical models that are suitable for better understanding the mechanisms of the welding process and avoiding the welding defects [12]. Modeling the hybrid laser-arc welding seems to be a reliable way to investigate the physical phenomena of the process [13]. In numerical simulation, it is important to ensure the accuracy of both thermal and mechanical analysis, which permits to evaluate the weldability and the structural deformation during the welding process [14].

The heat source is one of the critical issues in thermal simulation. Moreover, temperatures are the necessary data that can be employed to define residual stress and distortion in the welded plates. Correct calibration of the model is a prerequisite to establish good correlations between numerical and experimental results. Consequently, the accurate prediction of the temperature distribution during the welding process depends on the heat source model. Rosenthal (1946) was the first researcher who suggested a mathematical model for the heat source [15]. Since then, Dowden et al. (1983) proposed a three-dimensional mathematical model for the simulation of molten pool during laser beam welding [16]. For arc welding, the double-ellipsoidal power density model proposed by Goldak (1984) proved to be extremely efficient in the simulation of the heat source [17]. That movement of the heat source was described by Fourier’s law and the point, line, point and plane sources can be represented. One of the limits of Fourier’s law is the calculation of the transient temperature in the proximity of the fusion zone [18]. More recently, researchers have been struggling with the modeling of the laser-arc hybrid welding process. Reutzel et al. [19] used a volumetric double ellipsoidal heat source to represent laser/MIG (Metal Inert Gas) welding.

This paper presents a finite element model (FEM) for the thermal-mechanical simulation of the laser-arc hybrid welding process of DP/austenitic stainless steel (AISI 316) and TWIP/AISI 316 dissimilar steel welds. The heat input during the hybrid welding process was simulated by a three-dimensional moving heat source. This work focuses on the calibration and validation of the heat source by comparing the size and shape of the weld calculated by the numerical model and those obtained by the experiment. The metallurgical transformations for the DP steel were evaluated using the continuous cooling transformation (CCT) diagram and the calculated thermal cycle. This study is a novelty of the numerical modeling of a dissimilar steels weld obtained by a hybrid fiber laser-MAG welding system. Enhancing the weldability of dissimilar DP/AISI 316 and TWIP/AISI 316 steels can be a solution for the assembler of car body parts to reduce vehicle weight and, consequently, fuel consumption. Hence, it is important to have a model by which the welding parameters can be optimized.

## 2. Experimental Setup

**Figure 1:**
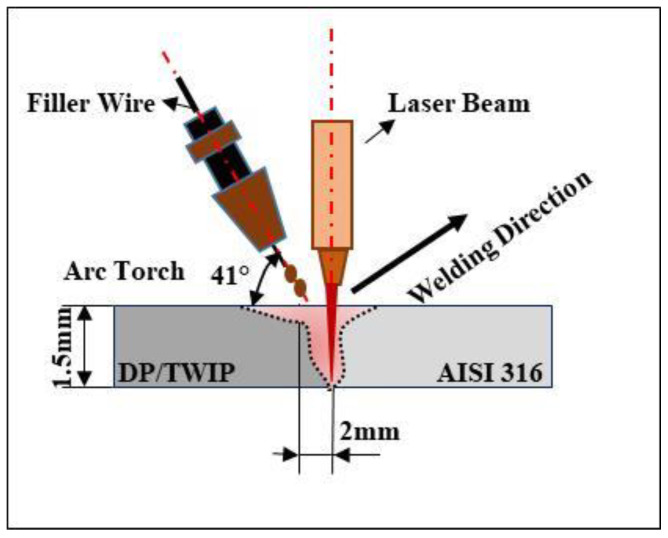
Layout of the hybrid laser-arc welding process.

Plates 1.5 mm thick of the DP/AISI 316 and TWIP/AISI 316 steels were welded in butt configuration, as shown in Figure 1. Table 1, Table 2 and Table 3 contain the chemical composition of those steels.

The welding system was a continuous ytterbium fiber laser (IPG YLS-4000) with power of 4 kW and wavelength of 1070.6 nm combined with a MAG (metal active gas) current generator. A mixture of argon (87%) and carbon dioxide (CO_2_) (13%) was used as shielding gas to avoid the melt pool oxidation during the welding process. The MAG torch, tilted at an angle of 41°, with 94 A arc current and 20.2 V voltage was used. The laser beam with 250 mm focal distance lens created a spot diameter of 0.4 mm on the workpiece surface. The welding parameters included 1500 W power at 2.4 m/min welding speed. Austenitic filler wire with a diameter of 0.8 mm was used (316L-Si/SKR-Si). Table 3 shows the chemical composition of the austenitic filler wire. As represented in Figure 1, the laser-leading configuration was used during the welding process. The heat sources were spaced by 2 mm. The laser focus was put at the sheet’s surface.

The cross section of the dissimilar joints was analyzed by optical microscopy (OM, Nikon Epiphot 200, Tokyo, Japan), after the standard metallographic grinding and polishing technique. The microstructure of the cross sections was etched using Glyceregia solution (15 mL HCl, 15 mL glycerol, 5 mL HNO_3_) for austenitic stainless steel and TWIP steel and LePera solution (1% metabisulfide in distilled water and 4% picric acid in ethyl alcohol) for DP steel.

## 3. Numerical Model

**Figure 2:**
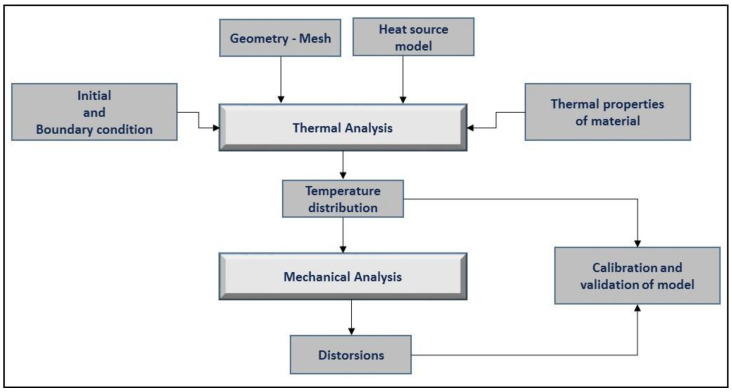
Flowchart of the simulation process.

The finite element model was built using the Simufact Welding software (version 8.0, MSC. Software, Munich, Germany). Thermo-mechanical finite element calculation was performed for the prediction of the fusion zone and prediction of the temperature fields, thermal cycles and distortions of the plates. The flow chart in Figure 2 describes the numerical procedure used in the FEM analysis: coupling thermal analysis was used as input for mechanical analysis step by step.

### 3.1. Geometry and Mesh

**Figure 3:**
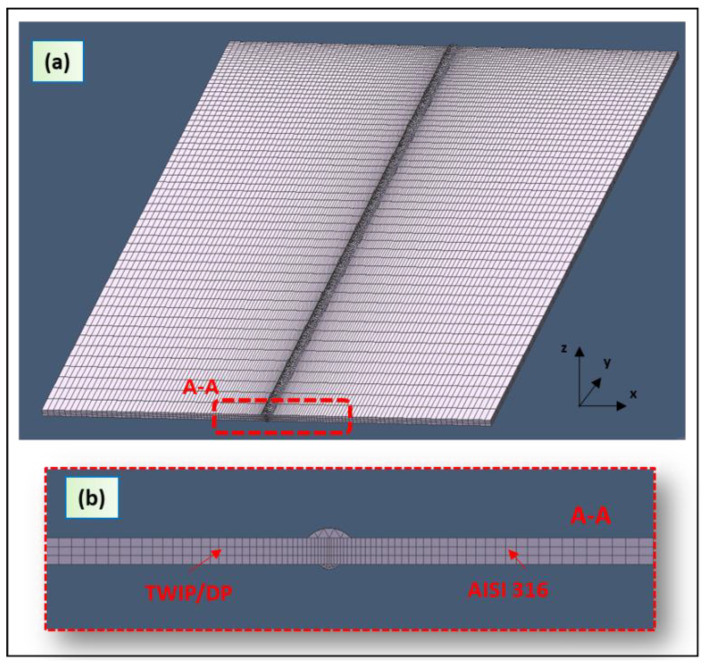
(**a**) Finite element model (FEM) used in the hybrid laser-arc welding; (**b**) side view of the model.

The dimensions of the plate were 150 mm × 50 mm × 1.5 mm. Hexahedral mesh was employed. Figure 3a shows the mesh used in the model. The fine mesh is very important for the accuracy of the temperature calculation, which is the most important precaution in high-thermal gradient zones.

Mesh sizes were 3.2 mm × 0.5 mm × 0.1 mm at the contact surfaces of the plates in order to reproduce the high thermal gradient. The ratio in size between the last element and first element in the distribution was 0.1 (Figure 3b). A coarse mesh was used far from the fusion zone to reduce the computational cost. The mapped mesh in the plats had 33,000 elements.

The top and bottom reinforcements produced during the welding processes requires a finer mesh. The tetrahedron mesh in the top and bottom material had 45,000 elements (see Figure 3b).

### 3.2. Material Properties

Table 4 shows the mechanical and thermo-physical properties at room temperature of DP, TWIP and stainless steel. Temperature dependent material properties were considered in the FEM analysis. The simulation strategy was to perform first the thermal analysis. The thermal simulation output was used for the mechanical analysis, which permitted the evaluation of the induced strain fields. 

### 3.3. Boundary Conditions

In the welding simulation process is fundamental the temperature distribution in the workpiece. Equation (1) accounts for the heat transfer in the plates during the welding process [25]:

(1) $$\frac{\partial }{\partial x}(K_{x}\frac{\partial T}{\partial x})+\frac{\partial }{\partial y}(K_{y}\frac{\partial T}{\partial y})+\frac{\partial }{\partial z}(K_{z}\frac{\partial T}{\partial z})+Q(x,y,z)=\rho c\frac{\partial T}{\partial t}$$

In this equation, *Q* (W·m^−3^) represents the heat transfer in the work piece and *ρ* (kg·m^−3^) the density of the metal. *K_x_*, *K_y_* and *K_z_* define the thermal conductivity in the *x*, *y* and *z* directions (W/m^−1^·K^−1^), *c* represents the specific heat capacity (J·kg^−1^ °C^−1^), while *t* is the time (s). 

Heat losses due to the convection and radiation were also considered. The heat losses followed Equation (2) [25]:

(2) $$-q=\varepsilon \sigma (T_{r}^{4}-T^{4})+h(T_{r}-T)$$

where ε is the emissivity of the surface, σ is the Stefan-Boltzmann constant, *T*_r_ is the room temperature (293 K), *h* is the heat transfer coefficient. It was estimated to 20 W/(m^2^·K) for air and 200 W/(m^2^·K) for the lower surface of the weld. The workpiece room temperature was 293 K at time t = 0. The boundary conditions in the mechanical analysis were applied in order to simulate the strain field. The plates were clamped to the worktable to prevent displacement: four edges of plates were fixed in order to avoid translation and rotation with appropriate clamping system.

### 3.4. Three-Dimensional (3D) Model for Laser Heat Source

Two different heat source models were defined and coupled to represent the arc and laser heat source in hybrid laser-arc simulation welding. A 3D model for the heat source was considered for both.

**Figure 4:**
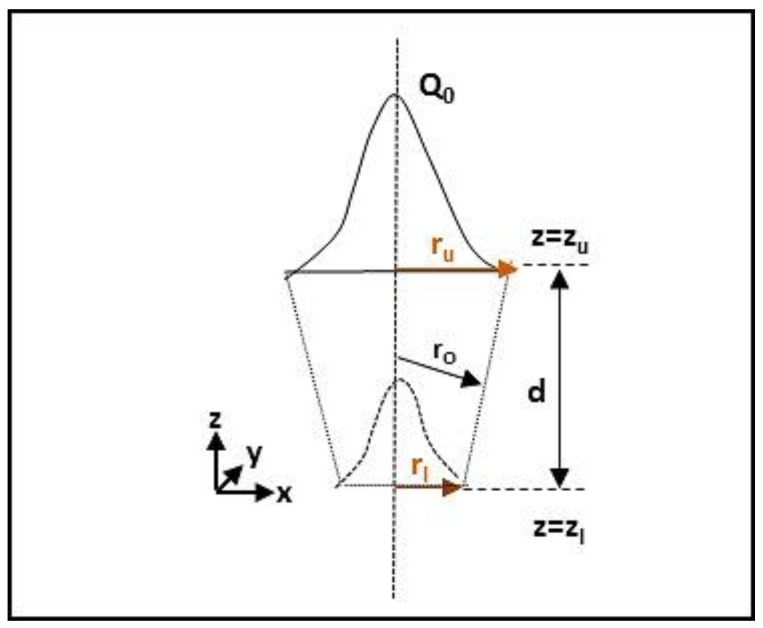
Conical heat source.

In the Gaussian distribution for the laser power density, the heat intensity decreases from the top to bottom of a truncated cone (Figure 4).

The power distribution in welding direction can be mathematically expressed as follows (Equations (3) and (4)) [26]:

(3) $$Q_{r}=Q_{0}\exp(-\frac{r^{2}}{r_{0}^{2}})$$

(4) $$r=\sqrt{x^{2}+y^{2}}$$

The parameter *r*_0_ decreases linearly and it can be expressed as Equation (5):

(5) $$r_{0}=r_{u}-\frac{\left(r_{u}-r_{l})(z_{u}-z\right)}{\left(z_{u}-z_{l}\right)}$$

In particular, *Q*_r_ represents the heat source intensity, *Q*_0_ is the maximum intensity, *r*_u_ and *r*_l_ describe upper and the lower radius in the upper plane at *z* = *z*_u_ and in the lower plane at *z* = z_l_ respectively.

### 3.5. Three-Dimensional (3D) Heat Source Model for the Electric Arc

**Figure 5:**
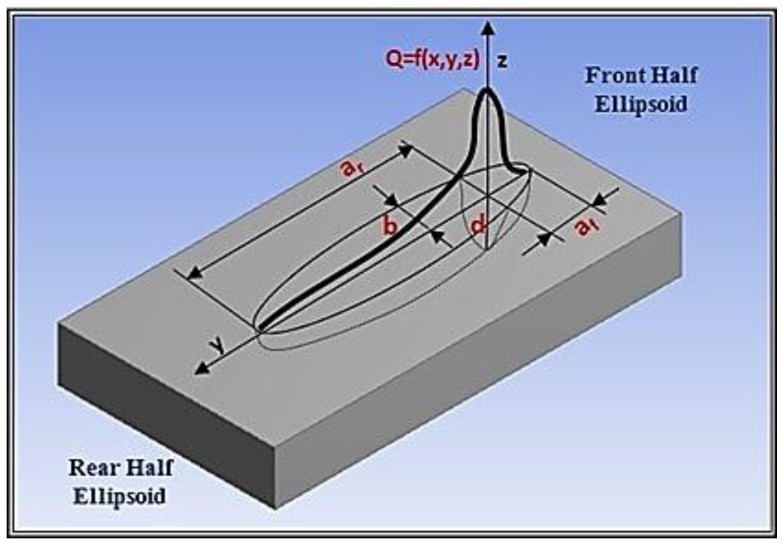
Goldak heat source distribution.

After Goldak, the double-ellipsoidal heat source can describe the arc power, which causes the molten pool [26]. The heat source model is shown in Figure 5; it combines two different ellipses, i.e., one in the front quadrant of the power distribution (*q*_f_) and the other in the rear quadrant (*q*_r_) [27]. 

Moving arc heat source in the plates can be mathematically defined as [28,29,30]:

(6) $$Q(x,y,z)=q_{f}(x,y,z)+q_{r}(x,y,z)$$

The mathematical definition of the power distribution of the two different ellipses is defined by power distribution inside the front quadrant (Equation (7)) and for the rear quadrant (Equation (8)), respectively.

(7) $$q_{f}=\frac{6\sqrt{3}f_{f}Q}{a_{f}bd\sqrt{\pi }}\cdot \exp(-\frac{3{\left(x-vt\right)}^{2}}{a_{f}^{2}}-\frac{3y^{2}}{b^{2}}-\frac{3z^{2}}{d^{2}})$$

(8) $$q_{r}=\frac{6\sqrt{3}f_{r}Q}{a_{r}bd\sqrt{\pi }}\cdot \exp(-\frac{3{\left(x-vt\right)}^{2}}{a_{r}^{2}}-\frac{3y^{2}}{b^{2}}-\frac{3z^{2}}{d^{2}})$$

In particular, *a*_f_ represent the front length, *a*_r_ is the rear length, *b* and *d* are the width and depth, respectively (see Figure 5). The welding speed is *v* and *f*_f_ and *f*_r_ are the proportion coefficients at front and rear ellipsoids. *Q* is arc power that was calculated by Equation (9):

(9) $$Q=\eta_{a}IU$$

where *I* is to the arc current and *U* represents the arc voltage and η_a_ is the arc heat efficiency. The heat source parameters are employed in the calibration process of the model.

**Figure 6:**
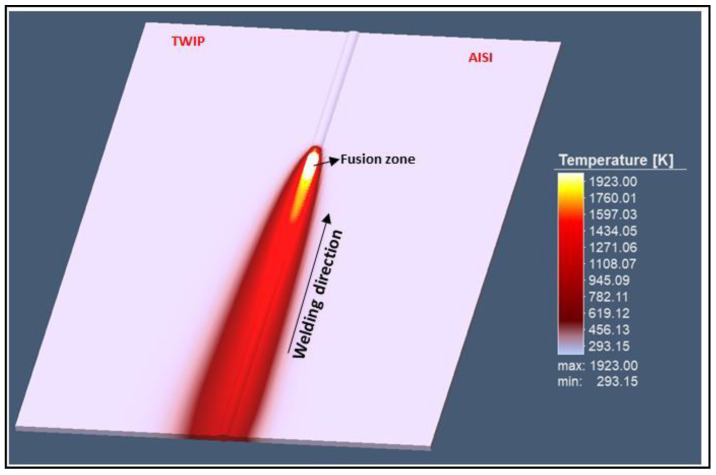
Temperature field during welding.

The temperature during the simulation analysis has reached 1923 K by ensuring the material fusion (Figure 6).

### 3.6. Mechanical Analysis

Thermal analysis results were input for the mechanical analysis. During the mechanical analysis, the same mesh size as during the thermal analysis was employed. Temperature-dependent mechanical properties of the material such as, Poisson’s ratio, Young’s modulus, and yield strength were used for the simulation.

The total strain is described by the following general Equation (10) [31,32]:

(10) $$\Delta \varepsilon ={\Delta \varepsilon }^{E}+{\Delta \varepsilon }^{p}+{\Delta \varepsilon }^{T}$$

where (Δε^E^) is the elastic strain, (Δε^p^)) is the plastic component and (Δε^T^) represents the thermal loading.

Another boundary condition assumed that the plates were clamped to the worktable to avoid translation and rotation. The buckling of the plates due to the expansion and contraction of the weld material during the welding processes was measured as the distortion.

## 4. Results and Discussion

### 4.1. Model Calibration

**Figure 7:**
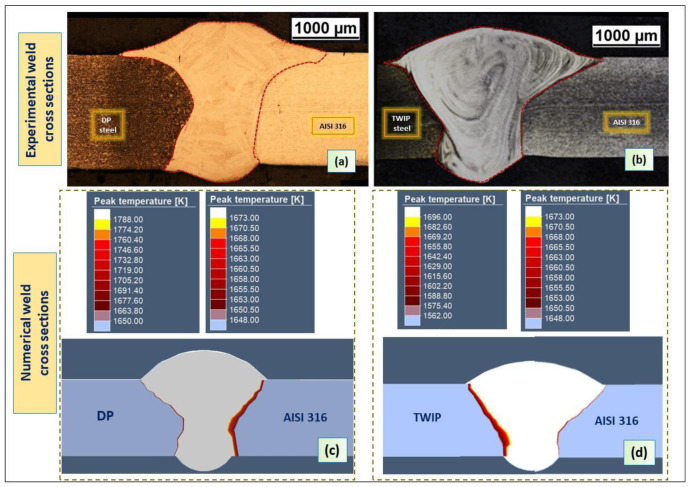
(**a**,**b**) Actual transversal sections; (**c**,**d**) numerical transversal sections.

The calibration of the heat source model was a trial-and-error process. The transverse section dimensions of the weld were the target of the calibration process. The calculated fusion zone was compared with that cross obtained during the experiments. Figure 7 shows the comparison between the simulated and experimental profiles for DP-AISI and TWIP-AISI welds, respectively. At the end of the calibration, the two geometries of the molten pool looked alike, which was the target of this preliminary phase of the simulation.

**Figure 8:**
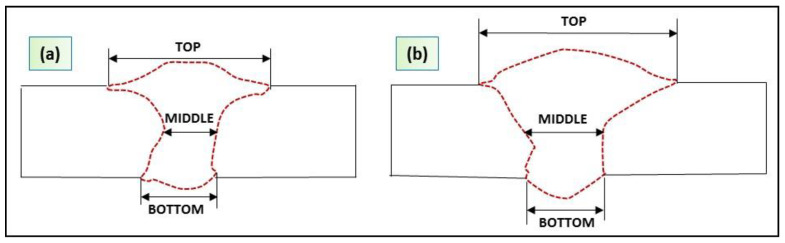
Weld cross section sketches: (**a**) DP-AISI 316 and (**b**) TWIP-AISI 316.

In order to predict the dimensions of the weld cross sections by the numerical model, the measurements were taken for their top, middle and bottom of joints. (see Figure 8). Dimensions of the fusion zones are given in Table 5 and Table 6. The numerical models for the two plates show good agreement between the predicted and the measured values.

The double-ellipsoidal and conical heat sources, which were used in the hybrid laser-arc welding simulation, provided a realistic modeling of the shape and dimensions of the fusion zone (Figure 7c,d).

In order to obtain a good fusion zone profile by the FEM analysis, the heat source parameters must be manipulated to calibrate the weld pool shape during the thermal-mechanical analysis. In particular, the conical heat source for laser was describe by three parameters, while the double ellipsoidal heat source for electric arc was defined through four parameters (Table 7 and Table 8). Table 7 and Table 8 show the parameters used to calibrate the models.

### 4.2. Model’s Validation

**Figure 9:**
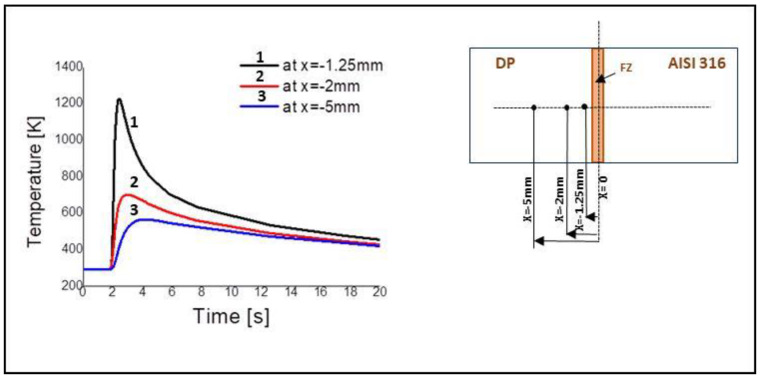
Thermal cycle of DP steels along the *x* direction: *x* = −1.25 mm, *x* = −2 mm, *x* = −5 mm.

The model was validated by comparison of the microstructure in the HAZ of DP. The microstructure was determined using the calculated cooling speed and the CCT curve. The cooling speed was measured at different points of the DP steel along the *x* direction. These points were located at −1.25 mm, −2 mm, −5 mm, respectively (Figure 9). During the FEM analysis, a high-temperature gradient occurred at the weld zone due to the interaction between the arc heating and laser energy. After the peak temperature, the welded plates cooled to room temperature. The thermal cycles are shown also in Figure 9.

**Figure 10:**
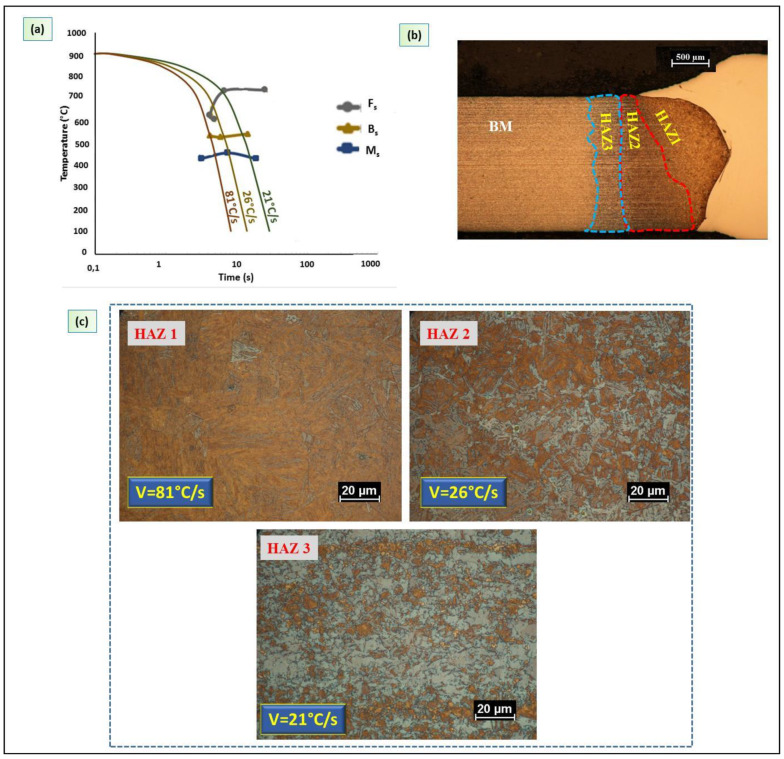
(**a**) Schematic representation continuous cooling transformation (CCT) curve for DP steel; (**b**) three sections of the heat-affected zone (HAZ); (**c**) details of HAZ and their cooling rate.

The phase transformation was checked through the cooling curves. DP steel exhibited a significant microstructural change in the heat-affected zone due to the thermal cycle during the welding process (Figure 10b,c). CCT are shown in Figure 10a [33]. Based on the CCT diagram, at high cooling rates (*v* = 81 °C/s), the formation of martensite with a small amount of bainite was figured out. Otherwise, a mix of ferrite banded martensite and bainite microstructure formed at slow cooling rates (26 °C/s and 21 °C/s) (Figure 10b,c).

The optical micrographs of the DP-AISI joint showed the effect of the cooling rate on the final microstructure. As a function of the distance from the fusion zone, the HAZ can be divided into three sections with different phases as follows:

(1) HAZ1—martensite with a small amount of upper bainite.
(2) HAZ2—banded martensite (according to the direction of rolling processing of sheet steel), as well as bainite and ferrite.
(3) HAZ3—ferrite with banded martensite.

**Figure 11:**
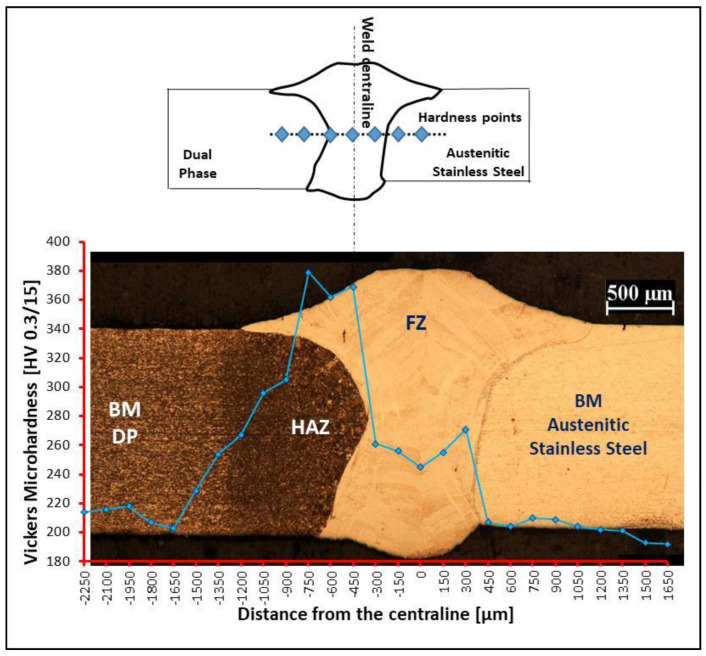
Micro-hardness test on transversal section of DP steel [3].

The microhardness of the transversal section confirmed the metallurgical transformations for DP steel (Figure 11). Due to the formation of martensitic and bainitic microstructures, the microhardness was as high as 380 HV.

**Figure 12:**
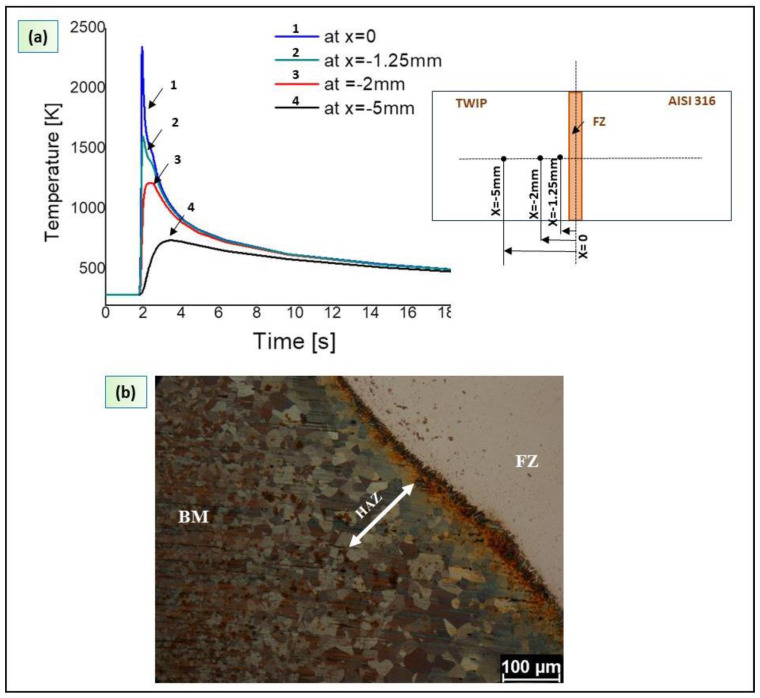
(**a**) Thermal cycles in different points of TWIP steel along the *x* direction; (**b**) grain coarsening in HAZ and fine austenitic microstructure in the base material.

Otherwise, a grain coarsening effect was observed in the HAZ of the TWIP steel. Figure 12a shows the thermal cycle measured in four different points along the *x* direction.

### 4.3. Weld Deformation

**Figure 13:**
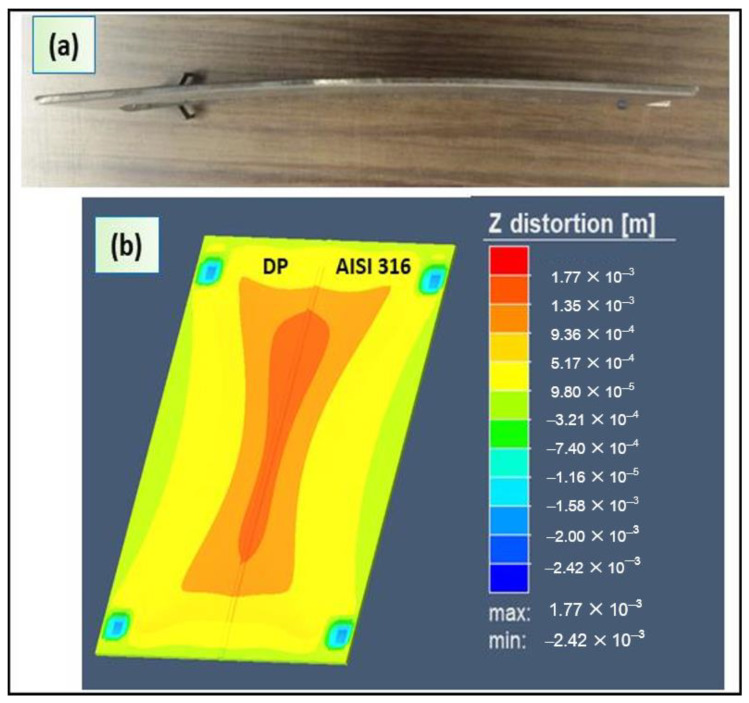
(**a**) Photo show distortion of the plate after welding; (**b**) simulation of welding residual distortion of the DP-AISI plate.

Furthermore, numerical and experimental distortions were checked for further validation of the process simulation. During hybrid laser welding, several types of deformation (bending distortion, longitudinal shrinkage, buckling or angular distortion) occurred due on the welding parameters and mechanical clamping conditions. During the welding process, large strain developed in the re-fused zone and its close regions. The plates showed a maximal distortion of about 2 mm (Figure 13a). This value was calculated using an image analysis.

The Figure 13b shows the simulated distortion values distribution in z direction after cooling and its comparison with the measured values. It was verified that the higher distortion existed at the fusion zone and around the HAZ (Figure 13b). Consequently, the conical and double-ellipsoidal heat source model adopted predicted distortion profile comparable with that of the measured distortion. 

## 5. Conclusions

Butt-welded joints of DP/AISI 316 and TWIP/AISI 316 were obtained by hybrid-arc laser welding. A 3D thermo-mechanical finite element model was developed to forecast temperatures, distortions and metallurgical transformation during the welding process. The model was validated by experimental data. The following results were obtained by means of the numerical simulation:

(1) The conical and double-ellipsoidal heat sources reproduced with accuracy the shape and dimensions of the fused and heat-affected zones.
(2) The phase transformations in HAZ of DP steel were reproduced and validated through the CCT curve. At high cooling rates, martensitic and bainitic microstructures formed, while at slow cooling rates a mixed microstructure was observed. In the HAZ of the TWIP steel, the grain was coarse.
(3) The model predicts accurately the distortions of the joint. The results achieved can be used to optimize the welding parameters with respect to weld distortions.
(4) The FEM simulation was in good agreement with the experimental data related to microstructure, weld shape, and distortion. Therefore, the presented model can be reasonably refined to sharpen its ability to predict the output of the hybrid welding process.

## Figures and Tables

**Table 1:** Chemical composition of twinning-induced plasticity (TWIP) and dual phase (DP) steels (wt %) [20].

**TWIP Steel**										
**C**	**Mn**	**Si**	**Al**	**Ni**	**Cr**	**Mo**	**V**	**Ti**	**Nb**	**Fe**
0.51	15.0	0.13	1	0.04	0.09	0.01	0.10	0.003	0.009	bal.
**DP Steel**										
**C**	**Mn**	**Si**	**Al**	**Ni**	**Cr**	**Mo**	**V**	**Ti**	**Nb**	**Fe**
0.09	1.65	0.21	0.03	-	0.43	-			-	bal.

**Table 2:** Chemical composition of austenitic stainless steel (AISI 316) stainless steel (wt %) [21].

AISI 316 Stainless Steel								
C	Cr	Mn	Mo	Ni	P	S	Si	Fe
0.08	18	2	3	14	0.045	0.03	1	bal.

**Table 3:** Chemical composition of austenitic filler wire (wt %) [20].

316L-Si/SKR-Si										
C	Mn	Si	Al	Ni	Cr	Mo	V	Ti	Nb	Fe
0.02	1.65	0.85	-	12	18	2.6	-	-	-	bal.

**Table 4:** Mechanical and thermo-physical properties at room temperature of DP, TWIP and AISI 316 steels [22,23,24].

Property	DP Steel	TWIP Steel	AISI 316
Young’s modulus (GPa)	235	171.5	205
Density (Kg/cm^3^)	7326	7500	7800
Thermal Conductivity (W/mK)	35.3	40	16.3
Liquidus Temperature (K)	1803	1648	1673
Solidus Temperature (K)	1713	1562	1643

**Table 5:** Table of experimental and numerical dimensions of the DP-AISI fusion zone.

DP-AISI	Experimental Data (mm)	Numerical Data (mm)	Errors (%)
Top	2.5	2.4	4.0
Middle	0.8	0.75	6.3
Bottom	1.1	0.8	27.3

**Table 6:** Table of experimental and numerical dimensions of the TWIP-AISI fusion zone.

TWIP-AISI	Experimental Data (mm)	Numerical Data (mm)	Errors (%)
Top	3.2	2.9	9.4
Middle	1.1	1.2	9.0
Bottom	1.1	1.2	9.0

**Table 7:** Parameters calibration of conical heat source for laser (see Figure 4).

Symbol	Reference	DP-Steel (mm)	TWIP-Steel (mm)
*r*_u_	Upper radius	0.3	0.6
*r*_l_	Lower radius	0.6	0.12
*d*	Depth	2.4	2.3

**Table 8:** Parameters calibration of double-ellipsoidal heat source for electric arc: DP and TWIP steel (see Figure 5).

Symbol	Reference	DP-Steel (mm)	TWIP-Steel (mm]
*a*_f_	Front length	0.36	0.55
*a*_r_	Rear length	1.5	1.9
*b*	Width	1.4	1.6
*d*	Depth	1.5	1
